# On the generalization and usability of cofolding models for GPCR drug discovery

**DOI:** 10.1038/s44386-026-00066-1

**Published:** 2026-08-14

**Authors:** Lichirui Zhang, Richard A. Friesner, Edward B. Miller, João P. GLM Rodrigues

**Affiliations:** 1 Schrödinger, Inc., New York, NY USA 1540 Broadway, 24th Floor, NY 10036; 2 Department of Chemistry, Columbia University, New York, USA 3000 Broadway, New York NY 10036

**Keywords:** Biochemistry, Biophysics, Chemistry, Computational biology and bioinformatics, Drug discovery, Structural biology

## Abstract

The generalizability of co-folding models for protein–ligand structure prediction remains unclear. Here, we benchmark Boltz, a state-of-the-art co-folding model, using a curated set of ligand-bound human G protein-coupled receptors (GPCRs) from families unseen during training. We show that while Boltz generally predicts receptor backbones accurately, ligand poses can contain significant errors that lead to a limited ability to reproduce experimental affinity data when tested with FEP +. We further show that physics‑based refinement of Boltz models can correct ligand poses to near‑experimental accuracy and rescue FEP+ performance to that of the native structure. These results highlight the strengths and limitations of co-folding methods and motivate a workflow that pairs them with physics-based refinement and validation before high-stakes decisions in drug discovery.

## Introduction

Deep learning has transformed computational structural biology. Models such as AlphaFold2^1,2^, RoseTTAFold^3^, and many others^4–8^ leverage co-evolutionary signals and, when available, structural templates to produce highly accurate protein structure models. Recent advancements, including AlphaFold3^9^, RoseTTAFold All-Atom^10^, Boltz^11,12^ and Chai-1^13^, extended the predictions to biomolecular complexes and enable end-to-end joint structure prediction of proteins with nucleic acids, metals, and small-molecule ligands. Most of these “co-folding” methods are diffusion-based generative models, which are intrinsically capable of producing multiple plausible conformations.

A central question, however, is to what extent these methods deliver robust and accurate predictions in real-world scenarios, such as drug discovery campaigns. Across multiple stages of drug discovery, decisions often hinge on model quality, and modeling errors can misdirect efforts, thereby incurring substantial time and financial costs. On standard benchmarks, deep learning methods appear impressive. For example, when evaluated on the PoseBusterV2 dataset^14^, a benchmark that monitors both the accuracy and physical plausibility of predicted poses, AlphaFold3 achieved an 80.5% success rate in predicting the native pose within 2 Å root-mean-square deviation (RMSD), rising to 93.2% when the binding pocket is specified. Yet, concerns persist regarding training set leakage and generalization to novel targets, an essential requirement in drug discovery, where ligands and, at times, targets, are often novel. Indeed, several studies suggest that co-folding models struggle to extrapolate beyond their training sets^15–18^, often produce unphysical structure models^19,20^, and struggle with placing ligands in allosteric binding pockets^21,22^, tempering expectations for immediate and broad applicability in drug discovery settings.

Traditional physics-based approaches, in contrast, offer a degree of robustness, generalizability, and interpretability owing to their grounding in well-established first principles. Rather than relying on probabilistic models of training distributions, they employ broadly applicable physical models to represent biomolecular interactions, generalizing naturally to new ligands and targets.

In this work, we aim to address three questions about co-folding methods through a physics-based lens, using Boltz-1x (hereafter “Boltz”), an open-source state-of-the-art model, as a representative co-folding method. First, do co-folding models generalize beyond their training sets? We evaluated Boltz on a curated benchmark of ligand-bound G protein–coupled receptors (GPCRs), a major class of drug targets. To better mirror prospective use in drug discovery, we restricted the benchmark to GPCR structures whose families were not included in the reported training set of Boltz. Second, are the predicted models fit for real-world decision-making? We assessed model usability with FEP + ^23^, a widely used free-energy perturbation (FEP) method for predicting ligand binding affinities. Because FEP+ is inherently sensitive to the finer details of its input structures, it can detect subtle yet consequential errors, such as misoriented ligand R-groups and misplaced receptor sidechains, that distance-based metrics like RMSD can average out. As such, failure to reproduce experimental binding affinities flags likely imperfections in the starting structures, a strategy employed in multiple prior studies^24–30^. Third, can such errors be remediated using physics-based approaches? We employ rigid-body and flexible-ligand docking methods, such as Glide^31^ or IFD-MD^32^, to refine Boltz models and test their impact on both pose and binding affinity prediction accuracy.

Overall, our results show that, in line with previously published observations, cofolding models struggle to produce accurate ligand poses that can effectively drive drug discovery. Furthermore, we demonstrate that structural or sequence similarity to the model’s training set does not reliably guarantee prediction quality. Instead, we demonstrate how free-energy perturbation can be used as a rigorous validation method for co-folding models in prospective scenarios, provided high-quality affinity data is available. Moreover, we show that co-folding models are a useful starting point for refinement with physics-based methods, and that such refinement can improve or, in some cases, completely rescue FEP+ performance of these models.

In summary, for maximal confidence, we advocate a workflow in which predicted structures seed physics-based refinement and validation before committing to design choices. This integrated paradigm leverages the speed of modern deep learning models while preserving the reliability required for decision-making in drug discovery. Ultimately, integrating co-folding models with physics-based techniques—whether during training, at inference, or sequentially—represents a highly promising frontier for future methodological development.

## Results and Discussion

Our major goal in this study was to evaluate how co-folding methods perform on tasks and metrics relevant to drug discovery, where experimental structures or close homologs might not be available. To this end, we assembled a benchmark of human GPCR complexes novel to Boltz, and assessed the resulting predicted models on the basis of RMSD to the deposited structure and on their ability to recapitulate binding affinity data with FEP + . We also tested whether physics-based methods, such as rigid-receptor (Glide) and induced-fit docking (IFD-MD), can help improve or rescue native-like ligand poses in cases where Boltz fails. Finally, we compared FEP+ performance starting from unrefined Boltz models versus IFD-MD–refined models, quantifying the impact of physics-based refinement on FEP+ accuracy.

### Docking performance of Boltz on GPCR dataset

We evaluated the accuracy of the top-scored Boltz model by calculating the receptor Cα and ligand heavy-atom RMSDs to experimental structures for the 253 GPCRs in our benchmark. While the transmembrane bundles of the receptors were accurately predicted (average Cα RMSD of 1.44 Å; SD 0.76 Å; median 1.25 Å), intra- and extracellular loop regions, as well as accessory domains were less accurate (average Cα RMSD of 2.90 Å; Sd 1.75 Å; median 2.63 Å). This result is expected and in line with other published reports^33^; the Boltz training set already contains several hundred GPCR structures that, even if evolutionarily distant, share the same conserved 7TM bundle fold.

Ligand predictions were, in contrast, considerably less accurate. The average ligand RMSD was 5.95 Å (standard deviation 7.03 Å; median 3.45 Å). If we defined the top N success rate as the top-N-ranked pose within 2.5 Å of the native ligand, which is a widely used threshold in the docking field, Boltz achieved top 1 and top 5 success rates of 37.15% and 44.66%, respectively. If the receptors are generally well predicted, what then drives poor ligand predictions?

**Fig. 1:**
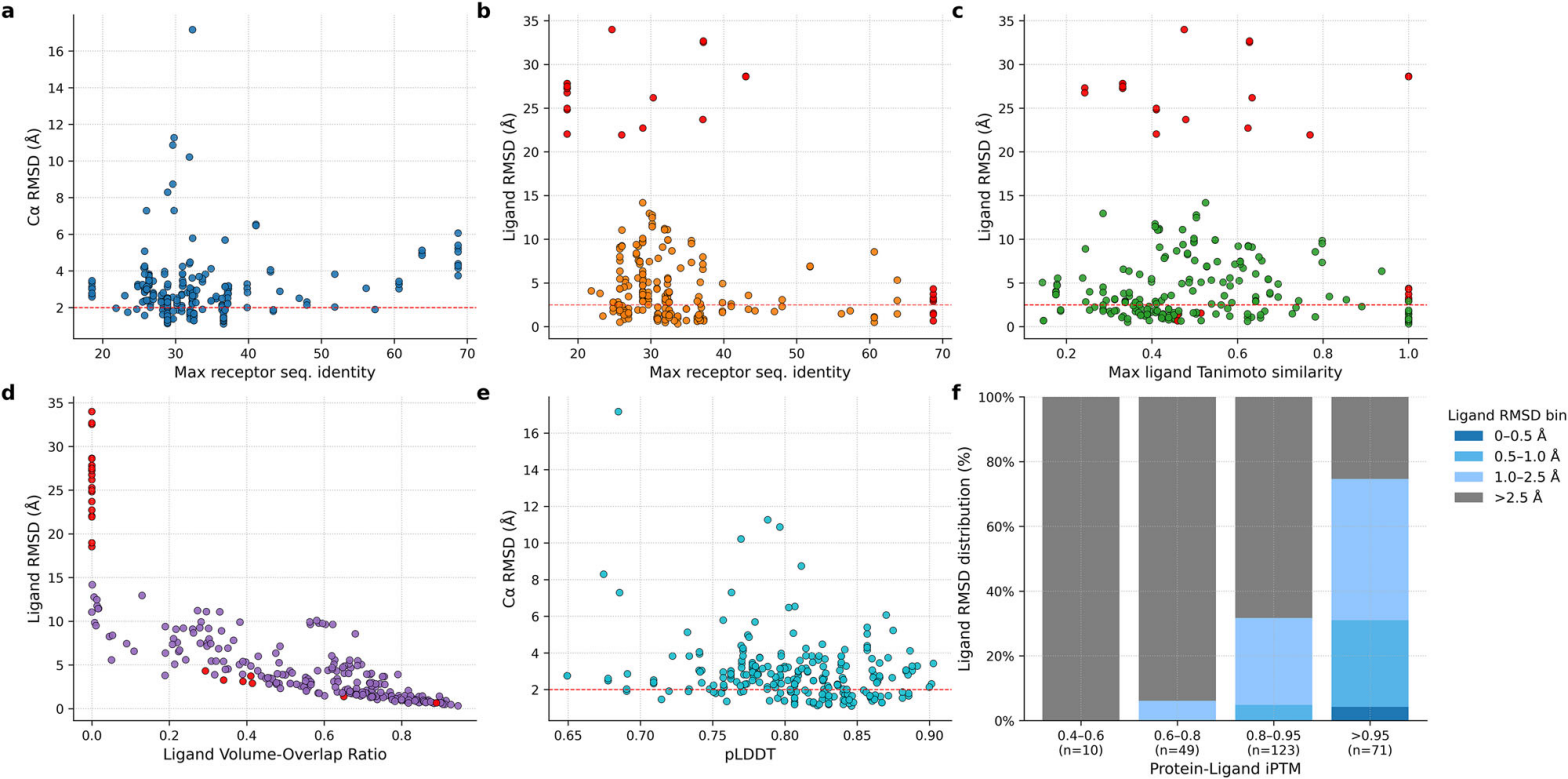
RMSD evaluation of Boltz models. **a** Cα RMSD vs. maximum receptor sequence identity to the training set. **b** Ligand RMSD vs. maximum receptor sequence identity. **c** Ligand RMSD vs. maximum ligand Tanimoto similarity. **d** Ligand RMSD vs. ligand volume-overlap ratio with the native ligand. **e** Average per-residue pLDDT vs. Cα RMSD. **f** Ligand RMSD vs. receptor–ligand ipTM. Allosteric ligand RMSD data points are highlighted in red for panels (**b**–**d**). Horizontal red dashed lines mark “success” thresholds at 2.0 Å for Cα RMSD and 2.5 Å for ligand RMSD, respectively.

First, and in agreement with previous studies^21,34,35^, we observe that Boltz performs poorly when predicting allosteric ligands, often forcing them into the orthosteric binding pocket (Fig. 1d, allosteric ligands highlighted in red). We hypothesize that this bias towards the orthosteric binding site arises from an imbalance in the model training set, although our analysis also shows that this error is particularly common among novel targets (Fig. 1b).

A second potential reason is the conformational plasticity of GPCRs, which is notoriously difficult to model with co-folding methods^33,36^. Co-folding methods tend to predict inactive receptor structures, while most of our dataset comprises agonists (228 out of 253 cases). To test this hypothesis, we re-predicted the complexes in our dataset and included the corresponding G-protein sequence (if present in the experimental structure). Across 201 paired complexes, while we observe substantial improvements to receptor model accuracy (Fig. S1a, median TM Cα improves from 1.01 Å to 0.75 Å), we do not observe significant improvement of ligand prediction accuracy (Fig. S1b, median improvement of 0.01 Å).

A third reason and common hypothesis is that the test set structures do not resemble the training set structures. However, we found no clear trend between the accuracy of receptor and ligand predictions and training set similarity (Fig. 1a–c). Boltz can produce very accurate models when both the receptor and ligand are dissimilar to the training data, while it can perform poorly when they are similar. Thus, for the cases in our benchmark, training set similarity showed little predictive value for pose quality, consistent with previous studies^15,37^. To further address this point, we re-ran the entire benchmark dataset with Boltz-2, an improved model that was released during the course of this work^12^. Boltz-2 was trained on an expanded dataset comprising structures deposited in the wwPDB before 2023-06-01, which includes 73 of our dataset cases. We show that while Boltz-2 produces more accurate ligand poses across the entire dataset (Fig. S2 and S3), the improvements are mostly concentrated among those structures that are part of the new model training set. For all other structures, there is no significant difference between Boltz-1x and Boltz-2 performance (*t* = 1.51, *n* = 180, *p* = 0.13; Wilcoxon signed-rank test: W = 7329, *p* = 0.2957).

### Confidence scores are useful markers of prediction quality

Our results show, however, that the confidence scores produced by Boltz are well calibrated to prediction accuracy, consistent with other deep learning methods^1,9^. As shown in Fig. 1f, a higher interface predicted TM-score (ipTM) generally corresponds to a lower ligand RMSD (R^2^ = 0.49), making ipTM a useful decision metric. Indeed, in our dataset, ipTM > 0.95 indicates a correct ligand pose (RMSD < 2.5 Å) for more than 75% of cases, whereas lower ipTM values indicate a need for additional validation or refinement. This confidence filtering is particularly valuable for detecting binding site errors. Cases where allosteric ligands were incorrectly placed into the orthosteric pocket consistently have ipTM scores below 0.80, suggesting that the model can potentially flag its own hallucinations regarding binding site location, preventing users from trusting these artifactual poses. However, we note that this observation does not extend to the predicted receptor accuracy score (pLDDT) (Fig. 1e; R^2^ = 0.046). A possible explanation is that our dataset is restricted to GPCRs, for which most receptor Cα positions are well-predicted even when the confidence score is low.

### Refining Boltz models with physics-based docking

We next asked whether physics-based methods could be used to rescue failed Boltz predictions. For the 253 top-scored GPCR Boltz models, we first filtered out the 114 cases where Boltz already produced a native-like ligand pose, defined as a ligand heavy-atom RMSD < 2.5 Å relative to the experimental pose. We then removed 55 “wrong pocket” cases where the predicted ligand center of mass was > 5 Å from that of the experimental ligand, since those cases fall outside the scope of refinement with most physics-based docking methods where knowledge of the binding site is a prerequisite. For the remaining 84 cases, we used Glide, a rigid-receptor docking method, to redock the ligands to the Boltz-predicted receptors. If there are severe errors in the receptor conformation, measured by clashes between the native pose and the predicted receptor, we expect Glide will be unable to recover the native pose. For these cases, we employed IFD-MD, an induced-fit docking method that has the potential to recover the native-like pose if there are only clashes between the native pose and the receptor sidechains or if limited amounts of backbone motion are required. Ultimately, for cases where the receptor backbone is poorly predicted, we did not expect either method to rescue the Boltz predictions. Although such cases could potentially be refined using long timescale simulations^38^, the computational cost and uncertainty in results led us to rule out such attempts.

**Fig. 2:**
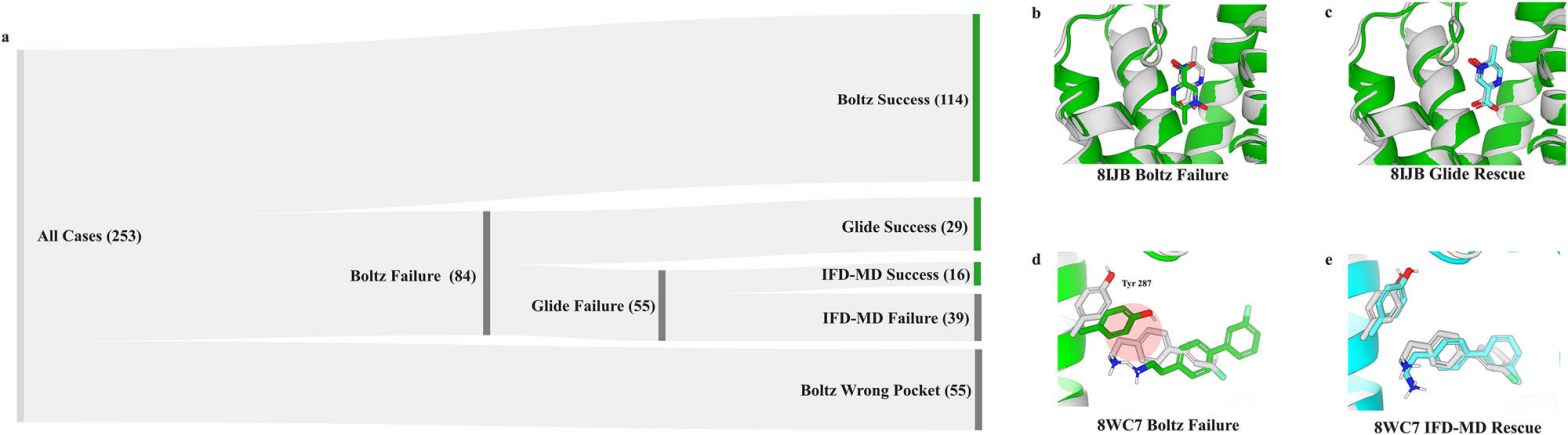
Hierarchical rescue analysis of Boltz predictions and physics-based refinement. Colors in panels (**b**–**e**): native (white), Boltz (green), model refined by physics-based methods (cyan). **a** Sankey plot showing the refinement pipeline and case numbers. **b** 8IJB Boltz failure. **c** 8IJB Glide rescue of a native-like pose. **d** 8WC7 Boltz failure. **e** 8WC7 IFD-MD rescue of a native-like pose. The Boltz receptor Tyr287 exhibits a steric clash with the native ligand pose. IFD-MD refines Tyr287 and recovers a native-like pose. Overall, Boltz succeeded in 114/253 (45.06%), failed in 84/253 (33.20%), and produced 55/253 (21.74%) wrong-pocket cases. Of the 84 Boltz failures, Glide recovered 29/84 (34.52%). IFD-MD further recovered 16/84 (19.05%). In total, physics-based docking methods recovered 45/84 (53.57%) cases.

Rigid-receptor docking with Glide recovered a native-like pose (RMSD < 2.5 Å) within the top 5 predictions in 29 of the 84 cases in which Boltz initially failed. IFD-MD then recovered a native-like pose within the top 5 predictions in an additional 16 cases, leaving 39 cases unrecovered by either method. In total, physics-based docking rescued 45 of the 84 (53.57%) of the cases in which Boltz failed, demonstrating that these methods can help refine Boltz-generated complexes. If we relax the criterion and consider the top 10 predictions for Glide and IFD-MD for each Boltz model, Glide rescued 36 of the 84 cases and IFD-MD recovered an additional 16, for a total of 52 of the 84 (61.90%) rescued cases (Fig. 2a). Although 10 poses per Boltz model is generous, as shown below, rigorous methods such as FEP+ can then be used to identify correct, high-quality predictions.

In Fig. 2, we highlight two examples of these physics-based refinement results. Figure 2b, c show that Glide successfully recovered a near-native pose (RMSD: 0.94 Å) from a flipped, incorrect Boltz pose (RMSD: 4.70 Å). In Fig. 2d, e, we demonstrate that, in the 8WC7 prediction, the receptor sidechain Tyr287 clashes with the native pose, making it unlikely that Glide could recover a native-like pose. By contrast, IFD-MD refined this clash to the native-like sidechain conformation, making room for the native-like ligand pose, and recovered a ligand pose with an RMSD of 1.21 Å.

We then examined why Glide and IFD-MD failed to rescue some Boltz predictions. Our hypothesis was that these models had major sidechain and backbone errors that prevented the native ligand pose from being docked correctly. To test this hypothesis, we quantified clashes between the native pose and the Boltz receptor models, defining a severe clash as any pair of heavy atoms closer than 0.75 times the sum of their van der Waals radii. Under this criterion, among the Boltz receptors where Glide failed to recover a native-like pose, 94.90% exhibit at least one severe clash with the receptor. A similar pattern was also observed for IFD-MD rescue failures, with 75.12% of the cases exhibiting at least one severe backbone clash (Fig. S4). This analysis, while limited by its retrospective character, highlights the robustness of physics-based methods in correctly predicting ligand poses given sufficiently accurate receptors. In prospective scenarios, as we show below, these methods benefit from guidance by rigorous scoring functions, such as FEP +.

Finally, we note that these results suggest a deeper synergy between physics-based refinement and deep learning methods that could outperform the current state-of-the-art and bring co-folding models into closer agreement with experimental structures.

### Assessing Boltz models as starting structures for FEP + 

One of the main uses of predicted structural models in drug discovery is to enable downstream structure-based design. Free energy perturbation methods, such as FEP + , offer an orthogonal assessment of model quality beyond RMSD. FEP + is a highly accurate physics-based method for predicting relative binding free energies that is sensitive to local errors in the initial structures. Previous studies^24–26^ have shown that predicted complexes with apparently acceptable ligand RMSDs can fail in FEP+ because subtle misplacements in the receptor and ligand can create large free-energy barriers that these methods cannot easily overcome. Such local yet consequential misplacements are not well captured by the RMSD metric, as well-predicted parts could average them out. Motivated by this, we evaluated whether Boltz models can serve as reliable starting structures for FEP+ calculations.

To gather benchmark data for validating Boltz structures with FEP + , we first needed to identify ligand series for which FEP+ could reliably recapitulate experimental structure–activity relationships using the experimental structures. Otherwise, if both the experimental and Boltz-predicted structures fail to produce a clear FEP+ signal, we cannot attribute the failure specifically to errors in the Boltz models, as it could also arise from limitations in the FEP+ protocol, uncertainties in the experimental affinities, or inaccuracies in the experimental structures themselves.

As such, we collected 27 congeneric series with experimental binding affinities targeting 14 GPCRs in our dataset. Refer to the Methods for details on the curation of this dataset. Of these, 14 series targeting 8 GPCRs exhibited a clear retrospective FEP+ signal, defined as R² > 0.3 and pairwise RMSE < 2.0 kcal/mol, while the remaining 13 did not (Table S1).

The absence of a clear FEP+ signal in the remaining 13 cases could be attributed to various factors, including subtle inaccuracies in the experimental structures in the initial ligand binding poses or receptor sidechain placements. For example, previous publications reported that the PDB structure 8 × 16 has poor map density in the extracellular loop region, suggesting potential uncertainties with sidechain and ligand modeling^39^. These uncertainties could be consequential in FEP+ calculations for certain ligand series. This also indicates that additional refinement, such as density-map-guided refinement, is probably necessary to obtain better FEP+ results^40^. Such issues could underlie many of the failures observed in our FEP+ validations for series targeting these experimental structures. A comprehensive analysis of these specific cases, however, is beyond the scope of this study.

We next ran FEP+ on the 14 ligand series that showed clear retrospective signals, using Boltz-predicted models as starting structures (Table S1). If the quality of the Boltz structures is comparable to that of the experimental structures, they should also show a comparable retrospective FEP+ signal. Our results show that, as expected, Boltz models in which the ligand was incorrectly modeled (7/14 series) failed to recapitulate experimental binding affinities in FEP + . The remaining 7 series could recapitulate, to varying degrees, the experimental structure FEP+ statistics. Two of these had poor starting ligand RMSDs (> 2.5 Å) and are discussed in detail below.

For Series 2 (ChEMBL ID: 615509; receptor PDB ID: 8IJD), a strong FEP+ signal arose despite a large initial pose RMSD (9.74 Å). Inspection of the FEP+ maps indicates that the high RMSD reflects a pose flipped by 180° around its long axis: the perturbations are centered on the central symmetric part of the ligands, and receptor interactions with those atoms are captured accurately (Fig. S5). Thus, FEP+ could perform well when interactions involving the perturbed portion of the ligand are correctly modeled, even if the global pose was incorrect. Series 10 (ChEMBL ID: 31833; receptor PDB ID: 8 × 17) also yielded a strong signal despite an initial RMSD of 3.05 Å. Here, experimental affinities correlate with the calculated octanol–water partition coefficient (AlogP; R² = 0.63), suggesting that hydrophobicity, rather than fine-grained receptor–ligand interactions, dominates affinity. These cases highlight the necessity of diverse chemical series targeting multiple sites on the molecule when using methods such as FEP + , to avoid drawing erroneous conclusions^28^.

### Impact of physics-based refinement on FEP+ performance of Boltz models

**Fig. 3:**
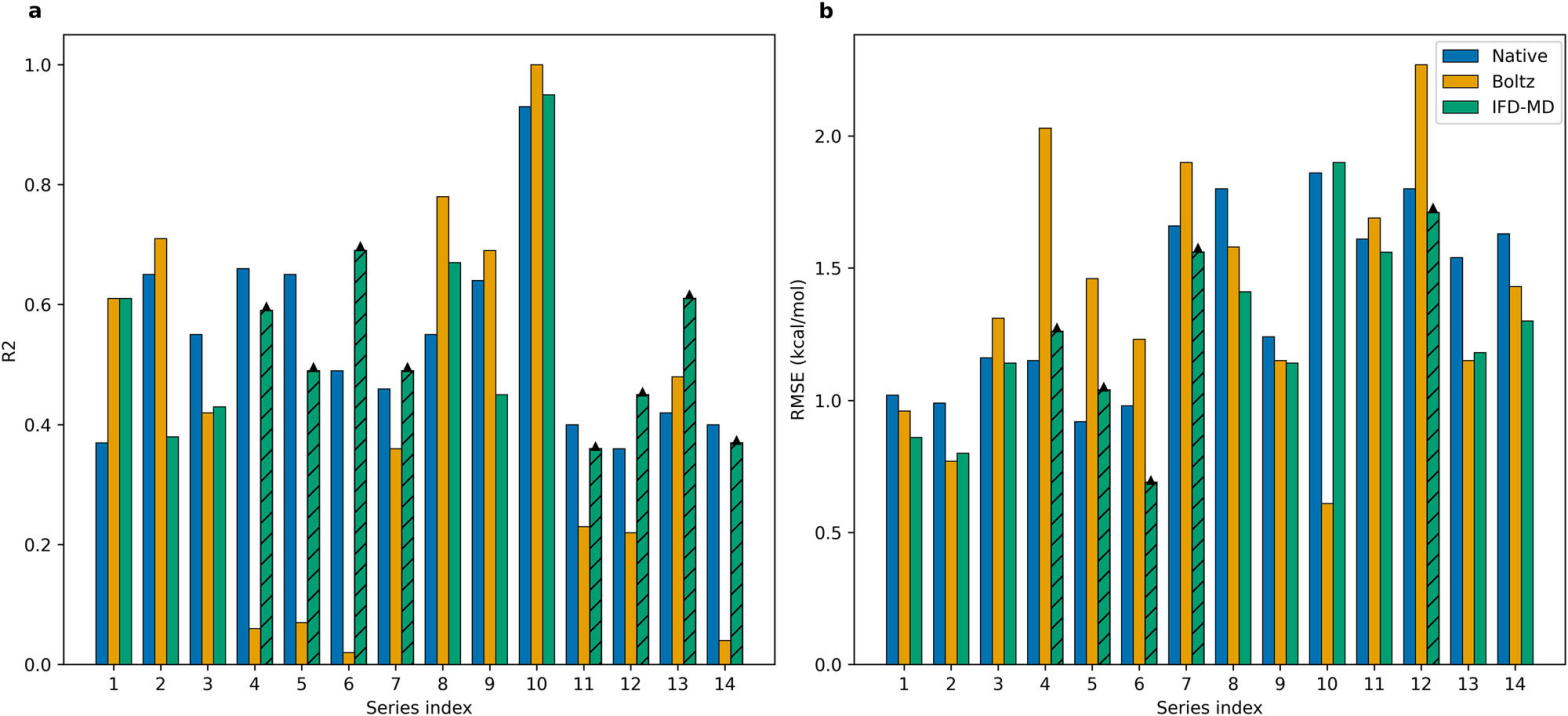
FEP+ performance of experimental structures, the best Boltz model, and the best IFD-MD models. **a** R². **b** RMSE. Hatched bars indicate IFD-MD results that outperform Boltz by a margin of 0.05 for R² and 0.2 kcal/mol for RMSE. A Wilcoxon signed-rank test was conducted for ΔR² = (R²_IFD-MD_ – R²_Boltz_) and ΔRMSE = (RMSE_IFD-MD_ – RMSE_Boltz_). The median ΔR² and ΔRMSE values are +0.095 and –0.150 kcal/mol, respectively, with corresponding i-values of 0.066 and 0.029. Cliff’s δ is 0.39 for ΔR² (95% CI –0.12 to 0.89, n_eff_ = 13) and 0.57 for ΔRMSE (95% CI 0.14 to 1.00, n_eff_ = 14), indicating a moderate to large tendency for IFD-MD to yield lower RMSE than Boltz and a non-significant trend toward higher R².

As shown earlier, IFD-MD showed potential to recover a native-like pose for Boltz structures in terms of ligand RMSD for cases where the overall receptor conformation was mostly correct. We had previously shown that IFD-MD can be used to refine AlphaFold2 models^25^, so we also assessed how IFD-MD affected FEP+ performance. To this end, we performed full IFD-MD refinement on all five Boltz models for each of the eight FEP + -validated receptors, then ran FEP+ validation on the top 5 IFD-MD poses from each model. As summarized in Table S1 and Fig. S6, except for Series 3, all thirteen remaining series contained at least one pose with ligand RMSD < 2.5 Å that also passed FEP+ validation (Fig. 3). By comparison, before IFD-MD, only five series met both criteria. These results show that physics-based methods like IFD-MD can consistently refine Boltz predictions, improving and, in some cases, rescuing FEP+ performance at moderate computational cost (average of 400 CPU-hours and 60 GPU-hours).

We highlight three cases for which none of the five Boltz models produced an FEP+ signal. First, for 8K2W, Boltz-predicted ligand RMSDs were above 5 Å for all poses, and none of the three related series showed an FEP+ signal. An IFD-MD-refined pose with an RMSD of 2.14 Å restored the FEP+ signal in all three series (Series 4 to 6) to near-native levels. Second, for 8 × 17, Boltz-predicted ligand RMSDs were higher than 3 Å and produced no FEP+ signal for Series 11 or 12. IFD-MD generated two native-like poses for Series 11 and three for Series 12, yielding FEP+ signals close to native performance. Third, for 8XVK, all Boltz ligand RMSDs were worse than 2.5 Å and none produced a strong FEP+ signal, whereas three IFD-MD-refined structures recovered a strong signal. In a prospective drug discovery setting, using the unrefined Boltz structures would not have enabled any of these targets, in contrast to IFD-MD refinement, which enabled all three. Finally, for series where Boltz predictions already produced an FEP+ signal, IFD-MD refinement did not degrade FEP+ performance significantly.

## Methods

### GPCR benchmark construction

We enumerated all human GPCR structures in GPCRdb^41^ that were initially released in the RCSB Protein Data Bank (PDB)^42^ after 2021-09-30 (the training cutoff for Boltz and AlphaFold3) and up to December 2024. We retained only receptors whose GPCR family, as defined by GPCRdb, had no structures released before that date, ensuring that the benchmark excludes families seen during training. It is worth noting that the definition of a “family” in GPCRdb accounts for the pharmacological classification of its endogenous ligands, rather than being purely based on sequence similarity^43^. We further required that the selected GPCRs had a small-molecule ligand bound, thereby excluding entries with protein and peptide ligands. This yielded 253 ligand-bound human GPCRs across 74 families as our GPCR benchmark (see Table S2 for a complete list). For each GPCR in the benchmark, we also identified the most similar sequence homolog and ligand among all GPCRs released before the training date cutoff, using BLAST^42^ and linear fingerprint Tanimoto similarity, respectively. Due to the family definition in GPCRdb, 8% of GPCRs in our benchmark have > 50% sequence identity with structures in the training set.

### Ligand series curation

To test whether FEP+ utilizing Boltz structural predictions can recapitulate experimental affinities, we obtained congeneric ligand series targeting the novel GPCRs from ChEMBL^44,45^. For each ligand-bound GPCR in the benchmark, we queried ChEMBL to find affinity measurements – activities – involving the ligand and the receptor. These activities were then grouped according to their source publication or patent, to ensure consistent experimental conditions^46^.

This search yielded 16,236 distinct measurements grouped into 798 ligand series matching a unique receptor-ligand-document triad, each of which is from a single assay. We then applied the following filters: (1) activities reported as K_d_, K_i_, or IC_50_ were retained because these values can be easily converted to binding free energies; (2) only measurements with an “equal” relation (excluding “>” or “<”) were retained; (3) the series must include the native ligand; (4) the series must contain more than five ligands with Tanimoto similarity > 0.5 to the native ligand; (5) each series must contain more than 12 ligands in total; and (6) series where > 80% of ligands overlap with another series were removed. Filter (1) allows a straightforward conversion between experimental affinity values and binding free energies. Filter (2) removes ambiguous or undefined measurements. Filters (3), (4), and (5) ensure suitability for FEP+ by favoring sufficiently large series with similar binding modes and small perturbations. Filter (6) avoids redundancy in calculations and analysis. After filtering, 27 congeneric series across 14 GPCR structures remained. All native ligands are bound in the orthosteric binding site of the receptors.

### Structure preparation

GPCR structures were downloaded from the PDB and prepared using the Schrödinger PrepWizard workflow^47^, which added hydrogen atoms, assigned protonation states to the ligand and titratable protein residues at the reported experimental pH, optimized hydrogen bonding networks, and performed a hydrogen-only minimization to avoid substantial deviations from the deposited structure. Except for the receptor chain and the native ligand, all other molecules were removed to reduce computational cost for FEP+ and IFD-MD, without harming the accuracy of subsequent physics-based calculations.

Ligand SMILES strings obtained from ChEMBL were converted to 3D coordinates using Schrödinger’s LigPrep. Ligand protonation/tautomeric states were generated at the reported experimental structure pH with Epik^46^. When ambiguity remained, we selected the state with the lower state penalty score reported by Epik. If ligand chirality was unclear, we consulted the original activity source paper. These prepared structures are available as MAE files in the Zenodo repository.

### FEP+ on experimental structures

To assess which GPCR–series pairs are amenable to retrospective FEP + , we first ran FEP+ on experimental structures. Initial poses for each series were generated with Glide^31^ using a maximum-common-substructure (GlideMCS) constraint to the native ligand. Ligands for which no acceptable pose could be found under the MCS constraint were excluded, potentially reducing series size from the original set. The structures were aligned to the corresponding structure in the Orientations of Proteins in Membranes (OPM) database^48^ for the membrane setup. The FEP+ simulations were run in the Schrödinger Suite (version 2025-2) using the OPLS4 force field^49^ and the SPC^50,51^ water model. Lambda scheduling depended on perturbation type: charge-changing perturbations used 24 windows, core-hopping perturbations used 16 windows, and all others used 16 windows. The total simulation time per leg was 50 ns, extended to allow predicted models to relax under simulation conditions. The Force Field Builder was used to generate missing torsion parameters for the ligands. All other parameters were left at defaults. Experimental activities were converted to binding free energies by assuming the temperature was 298 K. To minimize the FEP+ errors related to simulation time, we reanalyzed the FEP+ maps to 5 ns, 10 ns, and 25 ns, and picked the lowest RMSE result as the final result.

### Boltz predictions and FEP + 

Boltz-1x (v1.0.0) was obtained from GitHub. For each GPCR-ligand complex, we generated five diffusion samples using the full-length receptor sequence obtained from the UniProt database and the native ligand SMILES from ChEMBL. Multiple sequence alignments (MSAs) were generated using the Boltz default MSA server, with 10 recycling iterations per the recommendation in the Boltz technical report. Boltz’s predictions were prepared in the same manner as the experimental structures. To ensure consistency, the protonation/tautomer states of the ligand and protein titratable residues were forced to match the states in the prepared experimental structures.

For the same reason, the same FEP+ protocol and perturbation graph as the experimental structures were used for the Boltz FEP+ runs. For most of the 14 series, we found that there were a few ligands for which GlideMCS could not generate acceptable poses. This was due to clashes with the receptor sidechains or backbone under the MCS constraints, creating discrepancies with the ligand alignment performed in the native structure. As a result, we used the flexible ligand alignment method in the Schrödinger Suite, applying an MCS constraint to the Boltz-predicted pose, to align the ligands in each series and generate starting poses. The flexible ligand alignment method does not consider clashes with the receptor and so offers an alternative means of aligning poses compared with GlideMCS. Although some aligned poses initially clashed with the receptor, these were minor and easily resolved during the equilibration stages of FEP + .

### Glide and IFD-MD refinement and FEP + 

To test whether Glide and IFD-MD can improve or rescue Boltz predictions, each of the five Boltz diffusion samples for the GPCRs was redocked with Glide and IFD-MD. For Glide docking grid generation, the box center was placed at the centroid of the template ligand. For IFD-MD, the membrane protocol was used. All other settings were kept at their default values for both methods. For the five top-scoring IFD-MD structures, we ran FEP+ with the same protocol and perturbation graph as the experimental structures.

### RMSD calculations

We calculated Cα RMSD between predicted models and experimental structures by first aligning receptor structures via sequence alignment with the Biopython pairwise aligner, using the BLOSUM62 matrix and open and extend gap penalties set to 10 and 0.5, respectively. We then reported the resulting in-place Cα RMSD. For orthosteric ligand RMSD, to minimize errors introduced by aligning flexible intra- and extra-cellular receptor loop regions, we first aligned on transmembrane Cα atoms and then computed the in-place RMSD of ligand heavy atoms. Transmembrane (TM) residues were identified using the “Transmembrane” annotation in the UniProt database. For allosteric ligand RMSD, as the binding pocket often is far from the transmembrane bundle, we first aligned the binding pocket Cα atoms, defined as the Cα atoms within 10 Å of the native ligand, then calculated the ligand RMSD. The atom mapping between the native and predicted ligand structures was obtained using the FindMCS method in RDKit. Ligand symmetry, if present, was accounted for automatically during RMSD calculations.

## Supplementary information

Supplementary information

## Data Availability

The datasets generated and/or analyzed during the current study are available in a Zenodo repository (DOI: 10.5281/zenodo.18343531).

## Code availability

The underlying code for this study is available on GitHub and can be accessed via this link (https://github.com/schrodinger/Co-Folded-GPCR-Evaluation).
