# Supplementary material for: On the generalization and usability of cofolding models for GPCR drug discovery
Source: NPJ Drug Discov. 2026 Aug 14;3:30. doi: 10.1038/s44386-026-00066-1 (PMC13476267; doi:10.1038/s44386-026-00066-1)
Supplement: Supplementary file 1 [file 44386_2026_66_MOESM1_ESM.docx]

**Supplementary Information**

On the generalization and usability of cofolding models for GPCR drug discovery

Lichirui Zhang^1^, Richard A. Friesner^2^, Edward B. Miller^1^, João P.G.L.M. Rodrigues*^1^

^1^Schrödinger, Inc., 1540 Broadway, New York, New York 10036, United States

^2^Department of Chemistry, Columbia University, New York 10027, United States.

*Email: [joao.rodrigues@schrodinger.com](mailto:joao.rodrigues@schrodinger.com)

**Figure S1. Comparison of transmembrane bundle and ligand RMSDs from Boltz-1x predictions with and without inclusion of the G protein sequence.** (a) Transmembrane bundle RMSD. (b) Ligand RMSD. (c) Difference in the TM6 and TM7 (PDB ID: 8T3O). Colors in panel c: native (white), Boltz-1x prediction with (pink) and without (green) the G protein sequence. In some cases, G-protein inclusion placed the ligand in the correct binding pocket but in an incorrect pose, whereas in some other cases, ligands that should occupy the orthosteric pocket were incorrectly placed in the G-protein.

**Figure S2. Performance comparison between Boltz-1x and Boltz-2.** (a) Ligand RMSD. (b) Cα RMSD.

**Figure S3. RMSD Evaluation of Boltz-2 models.** (a) Cα RMSD vs. maximum receptor sequence identity to the training set. (b) Ligand RMSD vs. maximum receptor sequence identity. (c) Ligand RMSD vs. maximum ligand Tanimoto similarity. (d) Maximum receptor sequence identity vs. ligand Tanimoto similarity. (e) Ligand RMSD vs. ligand volume-overlap ratio with the native ligand. (f) Ligand RMSD vs. receptor–ligand ipTM. Non-orthosteric ligands are highlighted in red. Horizontal red dashed lines mark thresholds at 2.0 Å for Cα RMSD and 2.5 Å for ligand RMSD.

**Figure S4.** Distribution of backbone clashes between the Boltz receptor and native poses for IFD-MD refinement successes and failures.

**Figure S5. Series 2 Ligand Structures with their ChEMBL ID.** All the perturbations are centered on the ligand core. The Boltz-predicted pose is flipped 180 degrees perpendicular to its long axis, resulting in a 9.74 Å RMSD.

**Figure S6. IFD-MD redocking recovery.** The red dashed line denotes the 2.5 Å RMSD threshold for native-like poses. Among eight cases, five initially exhibited RMSD > 2.5 Å. IFD-MD refinement successfully recovered a native-like pose in four of these cases.

**Table S1. Retrospective FEP+ performance and starting-pose quality for each GPCR ligand series.** The FEP+ performance with the best RMSE among the Boltz and IFD-MD refined poses is reported. “Success rate” counts the number of ligands in the series whose starting pose RMSD is < 2.5 Å and that simultaneously show a clear FEP+ signal (R² > 0.3 and RMSE < 2.0 kcal/mol).

| **Series** | **PDB ID** | **Series ChEMBL ID** | **Ligand Pharmacology** | **# Ligands** | **Activity range (kcal/mol)** | **Native** | | **Boltz** | | | | **IFD-MD** | | | |
| --- | --- | --- | --- | --- | --- | --- | --- | --- | --- | --- | --- | --- | --- | --- | --- |
|  |  |  |  |  |  | **R^2^** | **RMSE (kcal/mol)** | **R^2^** | **RMSE (kcal/mol)** | **Ligand RMSD (Å)** | **# Success** | **R^2^** | **RMSE**  **(kcal/mol)** | **Ligand**  **RMSD (Å)** | **# Success** |
| 1 | 7UM4 | 1528152 | Antagonist | 19 | 1.37 | 0.31 | 0.91 | 0.61 | 0.96 | 0.75 | 5 | 0.61 | 0.86 | 0.73 | 8 |
| 2 | 8IJD | 615509 | Agonist | 10 | 3.29 | 0.75 | 0.81 | 0.71 | 0.77 | 9.74 | 0 | 0.38 | 0.8 | 2.04 | 1 |
| 3 | 8JXW | 366416 | Agonist | 14 | 3.48 | 0.55 | 1.15 | 0.12 | 1.49 | 7.85 | 0 | 0.43 | 1.14 | 8.63 | 0 |
| 4 | 8K2W | 438654 | Agonist | 24 | 5.17 | 0.65 | 1.12 | 0.06 | 2.03 | 7.05 | 0 | 0.59 | 1.26 | 2.01 | 1 |
| 5 | 8K2W | 619410 | Agonist | 19 | 3.52 | 0.65 | 0.93 | 0.07 | 1.46 | 6.43 | 0 | 0.49 | 1.04 | 2.01 | 1 |
| 6 | 8K2W | 799098 | Agonist | 17 | 1.87 | 0.53 | 0.49 | 0.02 | 1.23 | 6.78 | 0 | 0.69 | 0.69 | 2.14 | 1 |
| 7 | 8X16 | 303186 | Agonist | 18 | 3.01 | 0.46 | 1.64 | 0.36 | 1.90 | 2.30 | 3 | 0.49 | 1.56 | 1.85 | 8 |
| 8 | 8X16 | 31703 | Agonist | 18 | 3.81 | 0.55 | 1.79 | 0.78 | 1.58 | 2.40 | 4 | 0.67 | 1.41 | 1.87 | 8 |
| 9 | 8X16 | 349357 | Agonist | 12 | 4.20 | 0.64 | 1.23 | 0.69 | 1.15 | 2.3 | 4 | 0.45 | 1.14 | 1.87 | 7 |
| 10 | 8X17 | 31833 | Agonist | 7 | 8.07 | 0.93 | 1.93 | 0.90 | 2.45 | 3.05 | 0 | 0.95 | 1.90 | 2.13 | 2 |
| 11 | 8X17 | 439361 | Agonist | 14 | 2.50 | 0.55 | 1.5 | 0.23 | 1.69 | 3.05 | 0 | 0.36 | 1.56 | 2.23 | 1 |
| 12 | 8X17 | 522126 | Agonist | 24 | 6.00 | 0.52 | 1.68 | 0.22 | 2.27 | 3.23 | 0 | 0.45 | 1.71 | 1.93 | 2 |
| 13 | 8XVJ | 859881 | Antagonist | 57 | 5.70 | 0.42 | 1.53 | 0.48 | 1.15 | 1.69 | 4 | 0.61 | 1.18 | 0.90 | 14 |
| 14 | 8XVK | 65482 | Antagonist | 77 | 5.15 | 0.40 | 1.62 | 0.04 | 1.43 | 3.09 | 0 | 0.37 | 1.30 | 1.98 | 5 |

**Table S2.** Summary of the 253-GPCR benchmark used in this study. Max Lig. Sim. denotes the maximum ligand Tanimoto similarity to the Boltz-1x training set; Max Seq. Sim. denotes the maximum receptor sequence similarity to the Boltz-1x training set.

| PDB ID | Resolution | GPCR Class | GPCR State | Ligand CCD | Ligand Pharmacology | Max Lig. Sim. | Max Seq. Sim. |
| --- | --- | --- | --- | --- | --- | --- | --- |
| 8GW8 | 2.9 | Class B1 (Secretin) | Active | KHF | Agonist | 0.63 | 37.2 |
| 8IHI | 3.11 | Class A (Rhodopsin) | Active | P9X | Agonist | 0.37 | 28.88 |
| 8HOC | 3.0 | Class A (Rhodopsin) | Active | ITF | Agonist | 0.4 | 25.74 |
| 8JLK | 3.22 | Class A (Rhodopsin) | Active | UJL | Agonist | 0.34 | 32.51 |
| 8KFY | 3.06 | Class A (Rhodopsin) | Active | OXF | Agonist | 0.39 | 43.38 |
| 9IIW | 3.15 | Class T2 (Taste 2) | Inactive | A1AEI | Agonist | 0.33 | 18.52 |
| 8IY9 | 3.37 | Class A (Rhodopsin) | Active | NIO | Agonist | 0.18 | 28.88 |
| 8YKY | 2.99 | Class T2 (Taste 2) | Active | A1AEI | Agonist | 0.33 | 18.52 |
| 8WCC | 3.04 | Class A (Rhodopsin) | Active | HAI | Agonist | 0.19 | 32.51 |
| 8JII | 3.17 | Class A (Rhodopsin) | Active | NIO | Agonist | 0.18 | 28.88 |
| 8IYH | 3.3 | Class A (Rhodopsin) | Active | FI7 | Agonist | 0.5 | 28.88 |
| 8WCA | 3.48 | Class A (Rhodopsin) | Active | PEA | Agonist | 0.44 | 31.98 |
| 8W8S | 3.3 | Class A (Rhodopsin) | Active | U7D | Agonist | 0.33 | 22.2 |
| 8K5D | 3.74 | Class A (Rhodopsin) | Active | OKL | Agonist | 0.64 | 28.88 |
| 7XZ6 | 2.8 | Class A (Rhodopsin) | Active | I7J | Agonist | 0.58 | 26.94 |
| 8IYX | 3.34 | Class A (Rhodopsin) | Active | S6R | Antagonist | 0.47 | 32.28 |
| 8IYW | 3.45 | Class A (Rhodopsin) | Active | OKL | Agonist | 0.64 | 28.88 |
| 7UM4 | 2.8 | Class A (Rhodopsin) | Inactive | NN6 | Antagonist | 0.51 | 36.36 |
| 8HJ5 | 3.0 | Class A (Rhodopsin) | Active | U2U | Agonist | 0.48 | 28.01 |
| 8TF5 | 2.1 | Class A (Rhodopsin) | Active | OLA | Inverse agonist | 0.41 | 31.01 |
| 8JER | 3.45 | Class A (Rhodopsin) | Active | OJX | Agonist | 0.18 | 28.88 |
| 8J6R | 2.76 | Class A (Rhodopsin) | Active | FI7 | Agonist | 0.5 | 28.88 |
| 8W8A | 2.8 | Class A (Rhodopsin) | Active | T5U | Agonist | 0.31 | 31.98 |
| 8X79 | 2.41 | Class A (Rhodopsin) | Active | Y9Q | Agonist | 0.39 | 41.01 |
| 8ID4 | 3.1 | Class A (Rhodopsin) | Active | EIC | Agonist | 0.37 | 26.33 |
| 7XT9 | 3.2 | Class A (Rhodopsin) | Active | SRO | Agonist | 1.0 | 37.25 |
| 8G59 | 2.64 | Class A (Rhodopsin) | Active | YN9 | Agonist | 0.45 | 26.33 |
| 8KH5 | 2.83 | Class A (Rhodopsin) | Active | LPS | Agonist | 0.5 | 32.59 |
| 7UM5 | 2.73 | Class A (Rhodopsin) | Active | 8K3 | Agonist | 0.62 | 36.36 |
| 8YUT | 2.7 | Class A (Rhodopsin) | Active | A1D67 | Agonist | 0.47 | 30.95 |
| 8WC8 | 2.9 | Class A (Rhodopsin) | Active | VMT | Agonist | 0.37 | 31.98 |
| 8K5C | 3.13 | Class A (Rhodopsin) | Active | OJX | Agonist | 0.18 | 28.88 |
| 7XKE | 2.9 | Class B2 (Adhesion) | Active | AND | Agonist | 0.8 | 35.57 |
| 8YUV | 3.0 | Class A (Rhodopsin) | Active | A1LY4 | Agonist | 0.34 | 30.84 |
| 8DWG | 2.71 | Class A (Rhodopsin) | Active | U39 | PAM | 0.47 | 28.01 |
| 8YNT | 3.43 | Class A (Rhodopsin) | Inactive | A1D6T | Antagonist | 0.42 | 31.88 |
| 8HDP | 3.2 | Class A (Rhodopsin) | Active | ADN | Agonist | 1.0 | 60.64 |
| 8IJB | 3.23 | Class A (Rhodopsin) | Active | OJX | Agonist | 0.18 | 28.88 |
| 7XW5 | 2.96 | Class A (Rhodopsin) | Active | HOI | PAM | 0.53 | 51.83 |
| 7XP6 | 3.01 | Class T2 (Taste 2) | Active | SY9 | Agonist | 0.74 | 36.92 |
| 8K5B | 3.43 | Class A (Rhodopsin) | Active | NIO | Agonist | 0.18 | 28.88 |
| 8XVL | 3.22 | Class A (Rhodopsin) | Inactive | A1D5L | Antagonist | 0.54 | 63.79 |
| 8IHK | 3.21 | Class A (Rhodopsin) | Active | P9X | Agonist | 0.37 | 29.66 |
| 8T3O | 3.06 | Class A (Rhodopsin) | Active | YN9 | Agonist | 0.45 | 26.33 |
| 9D3G | 3.26 | Class A (Rhodopsin) | Inactive | EBX | NAM | 1.0 | 43.02 |
| 8K4N | 2.83 | Class A (Rhodopsin) | Active | VF0 | Agonist | 0.7 | 32.28 |
| 8KFX | 2.96 | Class A (Rhodopsin) | Active | OS9 | Agonist | 0.45 | 43.38 |
| 8IWM | 3.17 | Class A (Rhodopsin) | Active | PEA | Agonist | 0.44 | 34.87 |
| 8JXX | 3.06 | Class A (Rhodopsin) | Active | VCL | Agonist | 0.24 | 25.74 |
| 8IQ4 | 2.7 | Class A (Rhodopsin) | Active | 87Q | Agonist | 0.63 | 36.58 |
| 8I7V | 2.77 | Class A (Rhodopsin) | Active | OJX | Agonist | 0.18 | 28.88 |
| 8J6I | 2.92 | Class A (Rhodopsin) | Active | FI7 | Agonist | 0.5 | 28.88 |
| 7F58 | 3.1 | Class A (Rhodopsin) | Active | 1I8 | Agonist | 0.6 | 28.92 |
| 7XOV | 3.0 | Class A (Rhodopsin) | Active | IA1 | Agonist | 0.74 | 29.63 |
| 8I7W | 3.39 | Class A (Rhodopsin) | Active | OKL | Agonist | 0.64 | 28.88 |
| 8X2K | 3.03 | Class A (Rhodopsin) | Active | 5YM | Agonist | 0.24 | 28.22 |
| 8PM2 | 2.92 | Class A (Rhodopsin) | Active | 8IA | Agonist | 0.15 | 33.22 |
| 7YMJ | 3.35 | Class A (Rhodopsin) | Inactive | JGX | Antagonist | 0.41 | 36.78 |
| 8ID9 | 3.0 | Class A (Rhodopsin) | Active | EPA | Agonist | 0.32 | 26.33 |
| 7T6B | 3.19 | Class A (Rhodopsin) | Active | S1P | Agonist | 1.0 | 46.94 |
| 7XK2 | 3.1 | Class A (Rhodopsin) | Active | FI7 | Agonist | 0.5 | 28.88 |
| 8XQR | 3.2 | Class T2 (Taste 2) | Active | FLF | Agonist | 0.24 | 18.52 |
| 8JR9 | 2.57 | Class B1 (Secretin) | Active | KHF | Agonist | 0.63 | 37.2 |
| 8YN6 | 2.77 | Class A (Rhodopsin) | Active | ITF | Agonist | 0.4 | 30.84 |
| 7WCM | 2.33 | Class A (Rhodopsin) | Active | 8VP | Agonist | 0.43 | 26.94 |
| 8HDO | 2.87 | Class A (Rhodopsin) | Active | I5D | Agonist | 0.78 | 60.64 |
| 9IIX | 2.89 | Class T2 (Taste 2) | Active | A1AEI | Agonist | 0.33 | 18.52 |
| 8JHY | 2.87 | Class A (Rhodopsin) | Active | IX8 | PAM | 0.53 | 28.88 |
| 9JKQ | 2.66 | Class A (Rhodopsin) | Active | B40 | Agonist | 0.38 | 31.98 |
| 8IUK | 2.67 | Class A (Rhodopsin) | Active | UGU | Agonist | 0.65 | 36.58 |
| 8YNS | 3.33 | Class A (Rhodopsin) | Inactive | A1D6T | Antagonist | 0.42 | 31.88 |
| 8XQO | 2.77 | Class T2 (Taste 2) | Active | GOQ | Agonist | 0.41 | 18.52 |
| 8HQN | 3.0 | Class A (Rhodopsin) | Active | 9HO | Agonist | 0.29 | 29.8 |
| 7VUJ | 3.8 | Class A (Rhodopsin) | Active | 7ZQ | Agonist | 0.36 | 24.83 |
| 9IQT | 2.9 | Class A (Rhodopsin) | Active | NIO | Agonist | 0.18 | 28.88 |
| 8TRC | 3.3 | Class C (Glutamate) | Inactive | Z99 | Antagonist | 1.0 | 68.73 |
| 8XQT | 2.94 | Class T2 (Taste 2) | Active | CLR | Agonist | 1.0 | 18.52 |
| 8THK | 2.6 | Class A (Rhodopsin) | Active | CGZ | Agonist | 0.51 | 36.78 |
| 8ID6 | 2.8 | Class A (Rhodopsin) | Active | OLA | Agonist | 0.41 | 26.33 |
| 8ZSV | 2.96 | Class A (Rhodopsin) | Active | A1D8X | Agonist | 0.3 | 32.51 |
| 7W0M | 3.71 | Class A (Rhodopsin) | Active | 8EH | Agonist | 0.67 | 32.34 |
| 7UM6 | 2.79 | Class A (Rhodopsin) | Active | H8G | Agonist | 1.0 | 36.36 |
| 8JXW | 3.01 | Class A (Rhodopsin) | Active | VCF | Agonist | 0.43 | 25.74 |
| 8UXY | 3.3 | Class O2 (tetrapod specific odorant) | Active | XUQ | Agonist | 0.5 | 21.83 |
| 8TQB | 3.3 | Class C (Glutamate) | Active | W92 | Agonist | 0.46 | 68.73 |
| 8YRG | 3.14 | Class A (Rhodopsin) | Active | A1LZU | Agonist | 0.36 | 26.04 |
| 8WC7 | 3.1 | Class A (Rhodopsin) | Active | VMK | Agonist | 0.25 | 32.51 |
| 7SK7 | 3.3 | Class A (Rhodopsin) | Active | GJ9 | Agonist (partial) | 0.61 | 28.49 |
| 8XQP | 3.29 | Class T2 (Taste 2) | Active | GOQ | Agonist | 0.41 | 18.52 |
| 7UL3 | 3.0 | Class A (Rhodopsin) | Inactive | FO9 | Antagonist | 0.27 | 30.95 |
| 7YS6 | 3.0 | Class A (Rhodopsin) | Active | SRO | Agonist | 1.0 | 33.69 |
| 8H4I | 3.06 | Class A (Rhodopsin) | Active | HXA | Agonist | 0.29 | 26.33 |
| 8HNM | 2.94 | Class A (Rhodopsin) | Active | 4IE | Agonist | 0.33 | 37.13 |
| 8JLN | 3.24 | Class A (Rhodopsin) | Active | UJF | Agonist | 0.35 | 31.98 |
| 8IHJ | 3.07 | Class A (Rhodopsin) | Active | P9X | Agonist | 0.37 | 29.66 |
| 8IHH | 3.06 | Class A (Rhodopsin) | Active | P8A | Agonist | 0.18 | 28.88 |
| 8VHF | 3.51 | Class A (Rhodopsin) | Active | 8VP | Agonist | 0.43 | 26.94 |
| 8IUM | 3.14 | Class A (Rhodopsin) | Active | S2F | Agonist | 0.43 | 36.58 |
| 8ID8 | 3.0 | Class A (Rhodopsin) | Active | YN9 | Agonist | 0.45 | 26.33 |
| 8JIL | 3.5 | Class A (Rhodopsin) | Active | NIO | Agonist | 0.18 | 28.88 |
| 7S8N | 2.9 | Class A (Rhodopsin) | Active | 8IU | Agonist | 0.5 | 30.19 |
| 7YM8 | 2.92 | Class A (Rhodopsin) | Active | J5C | Agonist | 0.39 | 36.78 |
| 8IJA | 2.69 | Class A (Rhodopsin) | Active | NIO | Agonist | 0.18 | 28.88 |
| 7SK9 | 3.7 | Class A (Rhodopsin) | Active | GJ9 | Agonist (partial) | 0.61 | 28.49 |
| 8JLP | 3.23 | Class A (Rhodopsin) | Active | UJU | Agonist | 0.39 | 31.98 |
| 8IRV | 3.1 | Class A (Rhodopsin) | Active | R5F | Agonist | 0.41 | 57.33 |
| 8ZR5 | 3.31 | Class A (Rhodopsin) | Active | A1D8M | Agonist | 0.5 | 26.94 |
| 8YN4 | 2.97 | Class A (Rhodopsin) | Active | HSM | Agonist | 1.0 | 30.95 |
| 8HTI | 2.97 | Class O1 (fish-like odorant) | Active | OCA | Agonist | 0.33 | 23.42 |
| 8T3Q | 3.14 | Class A (Rhodopsin) | Active | HXA | Agonist | 0.29 | 26.33 |
| 8X16 | 3.29 | Class A (Rhodopsin) | Active | Q8L | Agonist | 0.89 | 48.0 |
| 7S8P | 2.6 | Class A (Rhodopsin) | Active | 8IX | Agonist | 0.41 | 26.04 |
| 7F8Y | 2.5 | Class A (Rhodopsin) | Inactive | 1OZ | Antagonist | 0.57 | 29.63 |
| 7XW6 | 2.78 | Class A (Rhodopsin) | Active | HOI | PAM | 0.53 | 51.83 |
| 8IHF | 2.97 | Class A (Rhodopsin) | Active | FI7 | Agonist | 0.5 | 28.88 |
| 7WCN | 2.87 | Class A (Rhodopsin) | Active | 8WL | Agonist | 0.58 | 26.94 |
| 8J6Q | 2.6 | Class A (Rhodopsin) | Active | 3HR | Agonist | 0.25 | 28.88 |
| 8SAI | 3.27 | Class A (Rhodopsin) | Active | S12 | Agonist | 0.7 | 32.28 |
| 8XZI | 2.7 | Class A (Rhodopsin) | Active | A1D5N | Agonist | 0.55 | 32.34 |
| 7YFD | 3.1 | Class A (Rhodopsin) | Active | ITF | Agonist | 0.4 | 25.74 |
| 8S4D | 2.58 | Class A (Rhodopsin) | Inactive | A1D5N | Agonist | 0.55 | 32.34 |
| 7SUS | 2.7 | Class A (Rhodopsin) | Inactive | 8EH | Agonist | 0.67 | 32.34 |
| 8XBI | 3.06 | Class A (Rhodopsin) | Active | KW3 | Agonist | 0.41 | 32.28 |
| 8WC6 | 3.2 | Class A (Rhodopsin) | Active | PEA | Agonist | 0.44 | 32.51 |
| 8J6P | 2.55 | Class A (Rhodopsin) | Active | NIO | Agonist | 0.18 | 28.88 |
| 8H4L | 3.07 | Class A (Rhodopsin) | Active | HXA | Agonist | 0.29 | 26.33 |
| 8JLO | 3.52 | Class A (Rhodopsin) | Active | UJL | Agonist | 0.34 | 31.98 |
| 7XTA | 3.2 | Class A (Rhodopsin) | Active | SRO | Agonist | 1.0 | 37.25 |
| 8XJL | 2.77 | Class A (Rhodopsin) | Active | UGU | Agonist | 0.65 | 36.58 |
| 8W88 | 2.6 | Class A (Rhodopsin) | Active | UJL | Agonist | 0.34 | 31.98 |
| 8IYS | 2.95 | Class A (Rhodopsin) | Active | YN9 | Agonist | 0.45 | 26.33 |
| 8HNL | 2.98 | Class A (Rhodopsin) | Active | 4AI | Agonist | 0.59 | 37.13 |
| 8I2G | 2.8 | Class A (Rhodopsin) | Active | O6F | Allosteric agonist | 0.73 | 56.13 |
| 7F8U | 2.8 | Class A (Rhodopsin) | Inactive | 1OE | Antagonist | 0.57 | 29.63 |
| 8XBE | 3.4 | Class A (Rhodopsin) | Active | KW0 | Agonist | 0.68 | 32.28 |
| 8HN8 | 3.0 | Class A (Rhodopsin) | Active | HSM | Agonist | 1.0 | 25.74 |
| 7XZ5 | 3.1 | Class A (Rhodopsin) | Active | LSC | Agonist | 0.81 | 26.94 |
| 8Y53 | 2.93 | Class A (Rhodopsin) | Active | A1D54 | Agonist | 0.46 | 32.89 |
| 8H2G | 3.01 | Class A (Rhodopsin) | Active | NIO | Agonist | 0.18 | 28.88 |
| 8YUU | 2.7 | Class A (Rhodopsin) | Active | HSM | Agonist | 1.0 | 30.84 |
| 8JSO | 3.4 | Class A (Rhodopsin) | Active | 1WE | Agonist | 0.39 | 31.98 |
| 8XOF | 2.6 | Class A (Rhodopsin) | Active | A1LVW | Agonist | 0.63 | 31.62 |
| 7XT8 | 3.1 | Class A (Rhodopsin) | Active | SRO | Agonist | 1.0 | 37.25 |
| 8W89 | 3.0 | Class A (Rhodopsin) | Active | PEA | Agonist | 0.44 | 31.98 |
| 9D3E | 3.02 | Class A (Rhodopsin) | Inactive | EBX | Allosteric antagonist | 1.0 | 43.02 |
| 8H4K | 3.1 | Class A (Rhodopsin) | Active | WPT | Agonist | 0.3 | 26.33 |
| 7SK8 | 3.3 | Class A (Rhodopsin) | Active | GJ9 | Agonist (partial) | 0.61 | 28.49 |
| 7XTC | 3.2 | Class A (Rhodopsin) | Active | 8K3 | Agonist | 0.62 | 32.78 |
| 8JEF | 2.96 | Class A (Rhodopsin) | Active | 3HO | Agonist | 0.29 | 29.66 |
| 7XKD | 2.4 | Class B2 (Adhesion) | Active | AND | Agonist | 0.8 | 35.57 |
| 7YAC | 3.24 | Class A (Rhodopsin) | Active | IUD | Agonist | 0.5 | 39.82 |
| 7B6W | 2.87 | Class A (Rhodopsin) | Inactive | T0B | Inverse agonist | 0.58 | 32.31 |
| 7VUH | 3.22 | Class A (Rhodopsin) | Active | 7ZQ | Agonist | 0.36 | 24.83 |
| 8XQN | 3.05 | Class T2 (Taste 2) | Active | GOQ | Agonist | 0.41 | 18.52 |
| 8G2Y | 3.44 | Class B2 (Adhesion) | Active | LPC | Agonist | 0.77 | 26.01 |
| 9IJA | 3.05 | Class T2 (Taste 2) | Inactive | A1AEI | Allosteric agonist | 0.33 | 18.52 |
| 8W87 | 2.8 | Class A (Rhodopsin) | Active | B40 | Agonist | 0.38 | 31.98 |
| 8YN3 | 2.56 | Class A (Rhodopsin) | Active | HSM | Agonist | 1.0 | 30.95 |
| 7XKF | 2.4 | Class B2 (Adhesion) | Active | AND | Agonist | 0.8 | 35.57 |
| 8YN5 | 2.7 | Class A (Rhodopsin) | Active | HSM | Agonist | 1.0 | 30.84 |
| 8TRD | 3.4 | Class C (Glutamate) | Inactive | Z99 | Antagonist | 1.0 | 68.73 |
| 7YK6 | 3.03 | Class A (Rhodopsin) | Active | IYF | Agonist | 0.5 | 28.29 |
| 8WCB | 3.1 | Class A (Rhodopsin) | Active | HAI | Agonist | 0.19 | 32.51 |
| 8J6J | 2.8 | Class A (Rhodopsin) | Active | OKL | Agonist | 0.64 | 28.88 |
| 8HNN | 3.6 | Class A (Rhodopsin) | Inactive | 43I | Antagonist | 0.48 | 37.13 |
| 8XQS | 3.3 | Class T2 (Taste 2) | Active | FLF | Agonist | 0.24 | 18.52 |
| 8JHN | 3.75 | Class A (Rhodopsin) | Active | UR9 | Agonist | 0.14 | 28.88 |
| 8J20 | 3.2 | Class A (Rhodopsin) | Active | 9T4 | PAM | 0.63 | 30.32 |
| 8DWH | 3.25 | Class A (Rhodopsin) | Active | U2U | Agonist | 0.48 | 28.01 |
| 7UM7 | 2.75 | Class A (Rhodopsin) | Active | H8D | Agonist | 1.0 | 36.36 |
| 9IJ9 | 2.7 | Class T2 (Taste 2) | Active | A1AEI | Allosteric agonist | 0.33 | 18.52 |
| 8X7A | 2.56 | Class A (Rhodopsin) | Active | Y9J | Agonist | 0.54 | 41.01 |
| 8YNA | 2.63 | Class A (Rhodopsin) | Active | A1LY4 | Agonist | 0.34 | 25.74 |
| 7EJX | 2.4 | Class A (Rhodopsin) | Active | J5F | Allosteric agonist | 0.48 | 24.66 |
| 8ITF | 3.46 | Class A (Rhodopsin) | Active | 8IA | Agonist | 0.15 | 34.87 |
| 8WW2 | 2.79 | Class A (Rhodopsin) | Active | OLA | Agonist | 0.41 | 28.22 |
| 8J19 | 3.23 | Class A (Rhodopsin) | Active | SWO | Agonist | 0.25 | 25.48 |
| 7WI6 | 3.71 | Class C (Glutamate) | Inactive | Z99 | Antagonist | 1.0 | 68.73 |
| 8T1V | 2.6 | Class A (Rhodopsin) | Inactive | XY8 | Inverse agonist | 0.43 | 31.01 |
| 8XBH | 2.83 | Class A (Rhodopsin) | Active | KW3 | Agonist | 0.41 | 32.28 |
| 8IW7 | 2.97 | Class A (Rhodopsin) | Active | PEA | Agonist | 0.44 | 34.87 |
| 8T1W | 3.49 | Class A (Rhodopsin) | Inactive | X7T | Inverse agonist | 0.55 | 31.01 |
| 8YN9 | 2.3 | Class A (Rhodopsin) | Active | HSM | Agonist | 1.0 | 25.74 |
| 8XBG | 3.43 | Class A (Rhodopsin) | Active | KW0 | Agonist | 0.68 | 32.28 |
| 8J22 | 3.2 | Class A (Rhodopsin) | Active | 9UJ | Agonist | 0.56 | 25.65 |
| 8UY0 | 3.2 | Class O2 (tetrapod specific odorant) | Active | 0WU | Agonist | 0.33 | 23.03 |
| 7YFC | 3.0 | Class A (Rhodopsin) | Active | HSM | Agonist | 1.0 | 25.74 |
| 8ID3 | 3.1 | Class A (Rhodopsin) | Active | 7NR | Agonist | 0.31 | 26.33 |
| 8JEI | 2.73 | Class A (Rhodopsin) | Active | CW3 | Agonist | 0.29 | 29.66 |
| 8TR2 | 3.5 | Class C (Glutamate) | Active | JIX | Agonist | 0.46 | 68.73 |
| 8JLR | 3.0 | Class A (Rhodopsin) | Active | VRK | Agonist | 1.0 | 31.98 |
| 8VY7 | 2.68 | Class T2 (Taste 2) | Active | A1AEI | Agonist | 0.33 | 18.52 |
| 8H8J | 3.2 | Class A (Rhodopsin) | Active | WYB | Agonist | 0.35 | 28.88 |
| 7S8O | 2.58 | Class A (Rhodopsin) | Active | 8IU | Agonist | 0.5 | 30.19 |
| 8POK | 3.4 | Class A (Rhodopsin) | Active | HSM | Agonist | 1.0 | 30.95 |
| 7YMH | 3.52 | Class A (Rhodopsin) | Active | E5E | Agonist | 1.0 | 36.78 |
| 7W0L | 3.57 | Class A (Rhodopsin) | Active | 8EH | Agonist | 0.67 | 32.34 |
| 7WIG | 2.7 | Class A (Rhodopsin) | Active | 9FI | Agonist | 0.59 | 39.82 |
| 8J6L | 3.05 | Class A (Rhodopsin) | Active | NIO | Agonist | 0.18 | 28.88 |
| 8JLZ | 3.09 | Class A (Rhodopsin) | Active | UQL | Agonist | 0.58 | 33.69 |
| 8J18 | 2.89 | Class A (Rhodopsin) | Active | HXD | Agonist | 0.29 | 25.48 |
| 8JXT | 3.07 | Class A (Rhodopsin) | Active | HSM | Agonist | 1.0 | 25.74 |
| 7VUG | 3.2 | Class A (Rhodopsin) | Active | 7ZQ | Agonist | 0.36 | 24.83 |
| 8WC4 | 3.1 | Class A (Rhodopsin) | Active | VMT | Agonist | 0.37 | 32.51 |
| 8ZRK | 2.82 | Class A (Rhodopsin) | Active | A1D8Y | Agonist | 0.43 | 26.94 |
| 8XJM | 2.85 | Class A (Rhodopsin) | Active | 7WT | Agonist | 0.42 | 36.58 |
| 8ZSP | 3.14 | Class A (Rhodopsin) | Active | 7LD | Agonist | 1.0 | 31.98 |
| 8K4S | 2.9 | Class A (Rhodopsin) | Active | JW0 | Agonist | 0.62 | 26.04 |
| 9DQH | 2.92 | Class A (Rhodopsin) | Active | A1BE2 | Agonist | 0.49 | 26.52 |
| 8THL | 3.1 | Class A (Rhodopsin) | Active | ALE | Agonist | 1.0 | 36.78 |
| 8VY9 | 2.88 | Class T2 (Taste 2) | Active | A1AEI | Agonist | 0.33 | 18.52 |
| 8XQL | 2.99 | Class T2 (Taste 2) | Active | GOQ | Agonist | 0.41 | 18.52 |
| 8XJK | 2.63 | Class A (Rhodopsin) | Active | A1D5A | Agonist | 0.45 | 36.58 |
| 8XIO | 2.65 | Class A (Rhodopsin) | Active | A1D5D | Agonist | 0.47 | 39.84 |
| 7XY6 | 2.99 | Class A (Rhodopsin) | Active | I5D | Agonist | 0.78 | 60.64 |
| 8RQL | 3.03 | Class T2 (Taste 2) | Active | FLF | Agonist | 0.24 | 18.52 |
| 8K2W | 3.0 | Class A (Rhodopsin) | Inactive | FI6 | Agonist | 0.65 | 37.13 |
| 8IHB | 2.85 | Class A (Rhodopsin) | Active | OKL | Agonist | 0.64 | 28.88 |
| 7XMT | 2.8 | Class A (Rhodopsin) | Active | I8B | Agonist | 0.46 | 44.28 |
| 8WC3 | 3.0 | Class A (Rhodopsin) | Active | UJL | Agonist | 0.34 | 32.51 |
| 7YK7 | 2.75 | Class A (Rhodopsin) | Active | IYM | Agonist | 0.6 | 28.29 |
| 8WC9 | 3.2 | Class A (Rhodopsin) | Active | VMT | Agonist | 0.37 | 32.51 |
| 8JLJ | 3.1 | Class A (Rhodopsin) | Active | UJF | Agonist | 0.35 | 32.51 |
| 8JLQ | 2.84 | Class A (Rhodopsin) | Active | G3C | Agonist | 1.0 | 31.98 |
| 8W8R | 3.3 | Class A (Rhodopsin) | Active | U7D | Agonist | 0.33 | 22.2 |
| 7VDH | 2.9 | Class A (Rhodopsin) | Active | 6IB | Agonist | 0.41 | 30.19 |
| 8X17 | 3.19 | Class A (Rhodopsin) | Active | XS0 | Agonist | 0.85 | 48.0 |
| 8YN8 | 2.77 | Class A (Rhodopsin) | Active | A1LY3 | Agonist | 0.33 | 30.84 |
| 7WIH | 3.68 | Class C (Glutamate) | Active | CWY | Agonist | 0.51 | 68.73 |
| 8JXV | 3.21 | Class A (Rhodopsin) | Active | VBU | Agonist | 0.42 | 25.74 |
| 7WI8 | 4.17 | Class C (Glutamate) | Inactive | Z99 | Antagonist | 1.0 | 68.73 |
| 8JZ7 | 2.6 | Class A (Rhodopsin) | Active | FI7 | Agonist | 0.5 | 28.88 |
| 8HVI | 3.04 | Class A (Rhodopsin) | Active | NFI | Agonist | 0.55 | 29.8 |
| 8UHB | 3.35 | Class A (Rhodopsin) | Active | WV8 | Agonist | 0.31 | 31.98 |
| 7VV6 | 3.3 | Class A (Rhodopsin) | Active | 6IB | Agonist | 0.41 | 30.19 |
| 8TB7 | 2.94 | Class A (Rhodopsin) | Active | ZOB | Inverse agonist | 0.62 | 28.9 |
| 7VV5 | 2.76 | Class A (Rhodopsin) | Active | 6IB | Agonist | 0.41 | 30.19 |
| 8ZX5 | 3.03 | Class A (Rhodopsin) | Active | A1D9D | Agonist | 0.94 | 25.75 |
| 7VUI | 3.3 | Class A (Rhodopsin) | Active | 7ZQ | Agonist | 0.36 | 24.83 |
| 7W41 | 2.95 | Class A (Rhodopsin) | Inactive | 8B8 | Antagonist | 0.61 | 34.08 |
| 8IQ6 | 3.4 | Class A (Rhodopsin) | Active | 7WT | Agonist | 0.42 | 36.58 |
| 7XY7 | 3.26 | Class A (Rhodopsin) | Active | NEC | Agonist | 1.0 | 60.64 |
| 7XTB | 3.3 | Class A (Rhodopsin) | Active | SRO | Agonist | 1.0 | 33.69 |
| 8TR0 | 3.2 | Class C (Glutamate) | Inactive | Z99 | Antagonist | 1.0 | 68.73 |
| 8IUL | 2.78 | Class A (Rhodopsin) | Active | 7WT | Agonist | 0.42 | 36.58 |
| 7XN9 | 2.6 | Class A (Rhodopsin) | Inactive | GI9 | Agonist | 0.66 | 39.82 |
| 8IJD | 3.25 | Class A (Rhodopsin) | Active | FI7 | Agonist | 0.5 | 28.88 |
| 8XVK | 3.21 | Class A (Rhodopsin) | Inactive | A1D5J | Antagonist | 0.42 | 63.79 |
| 9IYA | 3.04 | Class A (Rhodopsin) | Active | A1L3E | Antagonist | 0.66 | 25.75 |
| 7X5H | 3.1 | Class A (Rhodopsin) | Active | 8K3 | Agonist | 0.62 | 36.36 |
| 8KEX | 3.2 | Class A (Rhodopsin) | Active | JW0 | Agonist | 0.62 | 26.04 |
| 9DQJ | 2.9 | Class A (Rhodopsin) | Active | A1BEQ | Agonist | 0.49 | 26.52 |
| 8XVJ | 3.26 | Class A (Rhodopsin) | Inactive | A1D5I | Antagonist | 0.46 | 63.79 |
